# Gender-related differences on outcome following transcatheter mitral valve repair (TMVR): a systematic review and meta-analysis

**DOI:** 10.1186/s13019-023-02123-6

**Published:** 2023-01-17

**Authors:** Kang Yi, Jie Gao, Wen-Xin Wang, Yu-Hu Ma, Wei Wang, Shao E. He, Xiao-Min Xu, Peng-Fei Li, Tao You

**Affiliations:** 1 Department of Cardiovascular Surgery, Gansu Province, Gansu Provincial Hospital, No. 204, Donggang West Road, Lanzhou, 730000 China; 2 Gansu International Scientific and Technological Cooperation Base of Diagnosis and Treatment of Congenital Heart Disease, Lanzhou, China; 3 The First Clinical Medical College of Lanzhou University, Lanzhou, China; 4 The First Clinical Medical College of Gansu University of Chinese Medicine, Lanzhou, China; 5 The Second Clinical Medical College of Lanzhou University, Lanzhou, China; 6 Department of Pediatrics, First Affiliated Hospital of SunYat-sen University, Guangzhou, China; 7 Department of Neurosurgery, West China Hospital of Sichuan University, Chengdu, China; 8 Department of Cardiac Surgery, The First Hospital of China Medical University, Shenyang, China; 9 Division of Gastroenterology, Seventh Affiliated Hospital of Sun Yat-Sen University, Shenzhen, China

**Keywords:** Mitral valve regurgitation, Transcatheter mitral valve repair, MitraClip, Gender differences, Meta-analysis, Systematic reviews

## Abstract

**Background:**

The effect of gender on patients with mitral valve regurgitation (MR) undergoing transcatheter mitral valve repair (TMVR) remains to be defined. The aim of the present study is a comprehensive meta-analysis of studies that investigate differences between men and women after TMVR.

**Methods:**

A systematic literature search was carried out on eight databases to collect all relevant studies on gender-related outcomes of TMVR before March 1, 2021. The main outcomes of interest were mortality, cardiac function, MR class and other complications.

**Results:**

A total of eight literatures were included, all of which were retrospective observational studies. Compared to women patients, men had lower postoperative New York Heart Association (NYHA) class (OR = 1.53, 95%CI [1.23, 1.91], P = 0.0001) and higher incidence of postoperative acute kidney injury (AKI) (OR = 1.25, 95%CI [1.16, 1.34], P < 0.05). There were no significant difference on mortality in 30 days (OR = 0.95, 95%CI [0.81, 1.11], P = 0.53) and in 2 years (OR = 0.99, 95%CI [0.75, 1.30], P = 0.93), mitral valve regurgitation (MR) class (OR = 1.30, 95%CI [0.97, 1.75], P = 0.08) and incidence of myocardial infarction (MI) (OR = 0.88, 95%CI [0.65, 1.18], P = 0.38), stroke (OR = 0.80, 95%CI [0.63, 1.02], P = 0.08) and bleeding in hospital (OR = 0.84, 95%CI [0.59, 1.19], P = 0.32).

**Conclusions:**

Our meta-analysis demonstrates that men undergoing TMVR have worse preoperative diseases (diabetes mellitus, coronary artery disease, renal failure and myocardial infarction) while they have superior postoperative NYHA class at one-year. There are no significantly difference in other indexes between men and women.

## Introduction

Mitral valve regurgitation (MR) is a common heart valvular disease [1, 2] and affects 10% of people older than 75 years of age and 2% of the global population displays symptoms of mitral valve (MV) disease [3]. The rate of new diagnosis of MR (moderate or above) in people over 65 years old was 2.3%, which was much higher than that of aortic stenosis (0.7%) [4]. MR progresses in a concealed way, and serious consequences occur when it develops to decompensation of left ventricle function [5]. Moderate to severe MR is an important cause of heart failure [6]. MR includes primary MR caused by mitral valve disease and secondary MR caused by left ventricular function changes [7]. European Society of Cardiology, European Association for Cardio-Thoracic Surgery and American College of Cardiology had their recommendations of different treatments for MR [8, 9].

Surgical repair is the main treatment of MR [10], while some cases do not conform to its indications. Roughly half of the patients with severe symptomatic MR are not referred for surgery due to risks from age, comorbid factors, and frailty [3]. The 1 and 5—year mortality rates in these un-operated patients are 20–50% [11]. Transcatheter mitral valve repair (TMVR) can be used as one of the alternative treatment methods. In recent years, plenty of its devices have developed rapidly, such as MitraClip, PASCAL and Valve Clamp [12]. MitraClip is the most commonly used repair system and the only one certified by the US Food and Drug Administration (FDA), which has been proved to be safe and has shown good procedural success at > 90%, along with good long-term outcomes [3]. EVEREST I and II confirmed the validity and two landmark randomized trials, and COAPT and MITRA-FR further explored its indications [13–16].

Men and women have differences in pathophysiology, anatomical structure as well as the incidences of many basic diseases [17]. Men and women patients also have differences in MR. Compared with men, women have smaller left atrial volumes, ventricular volumes and absolute regurgitation [18]. When body size is taken as an indicator, the difference in heart size and regurgitation volume can be significantly reduced [19]. It has been reported that left ventricle reversal restructure after TMVR affects both men and women [20], and has been identified as an important prognostic factor [21]. Less women reach the recommended surgical criteria of ventricular enlargement in MR [8], which may result in worse outcomes after surgery [7]. Studies showed that men predominate in patients undergoing mitral valve or intervention and mitral valve surgery is beneficial for men [22, 23]. EVEREST II trial included 36.5% women and the COAPT trial included 33.4% women in the TMVR arm [22, 24]. It was different in the proportion of men and womenundergoing TMVR in our including studies, with women accounting for 41%. Pre and postoperative conditions of men and women are different. In recent years, more and more studies have reported postoperative conditions of TMVR based on gender differences. TMVR landscape is showing great promise going forward while there was not a systematic analysis of whether and how gender differences have an impact on the prognosis of TMVR. In this paper, the preoperative and postoperative status of TMVR caused by gender difference was comprehensively discussed, in order to provide a reference for clinical work.

## Methods

### Protocol and Registration

The protocol was registered on INPLASY (202,140,110) and is available in full on the inplasy.com (https://inplasy.com/inplasy-2021-4-0110). Ethical approval was not required for this work, because this was the analysis of previously published data.

### Literature search

The search strategy was identified which included terms: “gender” or “sex” or “sexuality” and “TMVR” or “PMVR” or “TMVI” or “PMVI or “transcatheter mitral valve repair” or “percutaneous mitral valve repair” or “transcatheter mitral valve intervention” or “percutaneous mitral valve intervention” or “transcatheter mitral valve edge-to-edge repair” or “percutaneous mitral valve edge-to-edge repair” or “MitraClip”. Then we searched eight databases including PubMed, the Cochrane Library, Web of Science, EMBASE, China Biology Medicine disc (CBM), China National Knowledge Infrastructure (CNKI), Wan Fang and VIP to retrieve all related studies before March 1, 2021. We also read the references of included studies to determine the additional supplement.

### Inclusion and exclusion criteria

Final selection included studies if they (1) reported patients with MR treated TMVR. (2) had available data and significant outcomes such as short- and long- term mortality and postoperative complications. (3) aimed to compare the differences between men and women. Then we excluded studies like abstract only, reviews, duplicates, animal research and other irrelevant studies. Two reviewers independently screened the literature according to inclusion and exclusion criteria, and cross-checked by reading the titles and abstracts of the obtained literature, the trials that did not meet the inclusion criteria were excluded, and then the full text of the suspected studies were read through to determine whether these studies were included. In case of disagreement, we consulted a third participant to assist in the judgment.

### Data extraction

According to the developed data extraction table, the data was extracted by using Microsoft Office Excel 2019. The extracted content mainly includes: (1) characters of the included research. (2) baseline of men and women. (3) outcome indicators: including mortality, postoperative complications and other items that can reflect the function of heart.

### Assessment of risk of bias in included studies

The risk of bias in the included literature was referenced to the Newcastle–Ottawa Scale (NOS) [25]. Evaluation items include: (1) Representativeness of the exposed cohort. (2) Selection of the non-exposed cohort. (3) Ascertainment of exposure. (4) Demonstration that outcome of interest was not present at start of study. (5) Comparability of cohorts on the basis of the design or analysis. (6) Assessment of outcome. (7) Was follow-up long enough for outcomes to occur. (8) Adequacy of follow up of cohorts. Only the fifth item was 2 points, other items were all 1 point. We assessed included studies and then gave integrated scores.

### Statistical analysis

All data analysis was performed using RevMan5.3 software. Binary variables are represented by odds ratio (OR), continuous variables are represented by mean difference (MD) for consistent measurement units. All variables are calculated with 95% confidence intervals (95%CI). All reported P values are two-sided, and P < 0.05 is considered statistically significant. Heterogeneity test was performed on the included studies using Q test and I^2^ test. The fixed effect model was used for analysis only when P > 0.10 or I^2^ ≤ 50%. Otherwise, the heterogeneity of the study was considered significant and the random effects model was used for analysis.

## Results

### Literature search and study selection

As shown in Fig. 9, initial screening retrieved 775 articles and 73 were excluded for duplication. After extracted the exclusion criterion above, we analyzed 22 potentially relevant studies. We then eliminated 14 studies for unavailable data, non-related, reviews and no-comparison. Eight studies [26–33] were finally included which enrolled 25,736 patients (12,104 men and 13,632 women) and finally analyzed for our meta-analysis.

### Study and patients’ characteristics

We present the characteristics of these studies in Table 1. Major items like the first author, the year of publication, the country and region of the research object, the resources of data, the duration of the research were finally shown. All studies were cohort studies. Four studies definitely reported MitraClip system approach. Data of four studies were come from different clinical databases and other data were resulted from hospitals or study centers.

**Table1:** Baseline characteristics of individual studies and patients

First Author	Year	Region	Study time	Patients			Follow-up time	Age (Years)	
				All (n)	Men (n)	Women (n)		Men (n)	Women (n)
Attizzani	2015	Italy	2008.08–2013.12	171	106	65	30d,12 m	70.29 ± 10.32	74.02 ± 8.51
Rodrigo	2015	US	2009.08–2012.11	173	109	64	30d,6 m	73.5 ± 10.2	78.9 ± 8.8
Gafoor	2016	Germany	2008.10–2011.04	567	362	205	30d,12 m	72 ± 10	76 ± 8
Tigges	2016	Germany	2008.09–2015.04	592	362	230	12 m,24 m	74.4 ± 8.4	76 ± 9.2
Doshi	2018	US	2012.01–2014.12	521	302	219	–	73.3 ± 15	72.8 ± 14.1
Elbadawi	2020	US	2012–2016	10,015	5300	4715	–	76.08 ± 12.095	76.30 ± 12.296
Muhammad	2020	UK	2010–2017	15,264	8080	7184	–	–	–
Werner	2020	Germany	2010.08–2013.07	828	501	327	30d,12 m	74.2 ± 8.5	77 ± 8.3

BMI		Logistic EuroSCORE		STS score		Hypertension		Diabetes mellitus		Prior PCI		Prior CABG	
Men (n)	Women (n)	Men (n)	Women (n)	Men (n)	Women (n)	Men (n)	Women (n)	Men (n)	Women (n)	Men (n)	Women (n)	Men (n)	Women (n)
–	–	7.68 ± 6.5	7.98 ± 7.21	6.37 ± 6.91	6.78 ± 7.13	79	48	36	24	34	20	–	–
–	–	18.3 ± 14.3	19.4 ± 16.1	–	–	66	41	28	7	31	10	37	8
26.4 ± 4	25.6 ± 5.1	22.7 ± 18.1	23.6 ± 18.7	–	–	–	–	–	–	–	–	–	–
25.8 ± 4.1	25 ± 4.9	–	–	–	–	254	170	119	45	166	72	146	33
–	–	–	–	–	–	207	142	84	53	–	–	–	–
–	–	–	–	–	–	3590	3200	1430	1125	1040	600	1725	665
–	–	–	–	–	–	4043	3488	894	700	1546	895	2428	939
26.7 ± 10.4	26.5 ± 15.1	24.7 ± 16.9	24.2 ± 20.6	8.9 ± 8.5	7.7 ± 5.6	324	200	139	109	–	–	158	43

Prior stroke		Coronary Artery disease		COPD		Renal failure		Atrial Fibrillation		Previous MI		NYHA ≥III		MR ≥ 3 grade	
Men (n)	Women (n)	Men (n)	Women (n)	Men (n)	Women (n)	Men (n)	Women (n)	Men (n)	Women (n)	Men (n)	Women (n)	Men (n)	Women (n)	Men (n)	Women (n)
8	6	–	–	23	14	46	36	45	22	45	13	85	54	104	62
12	3	–	–	15	17	–	–	50	28	50	13	103	62	–	–
25	10	264	90	79	28	167	70	246	137	138	43	294	172	355	199
66	28	266	115	79	41	230	105	250	148	145	50	344	214	362	228
–	–	–	–	–	–	–	–	–	–	40	27	–	–	–	–
625	560	3720	2525	–	–	–	–	–	–	–	–	–	–	–	–
–	–	5457	3696	–	–	3470	2170	–	–	–	–	–	–	–	–
58	23	250	108	119	65	–	–	203	151	151	69	430	285	–	–

We determine our baseline of men and women including age, body mass index (BMI), Logistic Euro SCORE, STS score, New York Heart Association (NYHA) heart function class (Class I: No limitation of physical activity; Class II: Slight limitation of physical activity; Class III: Marked limitation of physical activity; Class IV: Unable to carry out any physical activity without discomfort), MR class (Class 1: none; Class 2: mild; Class 3: moderate; Class 4: severe), major preoperative disease and operations and made a comparability analysis as shown in Table 2, which showed that compared to women, men had higher BMI and higher rate of diabetes mellitus, coronary artery disease (CAD), renal failure, myocardial infarction (MI), but younger than women. Except these items, others have no significant differences between men and women.

**Table 2:** Baseline comparability analysis results

Baseline	Studies	Data type	Effect measure	Model	Participants	Effect estimate	LCI	UCI	Q	P(Q)	Z	P(Z)
Age (Years)	7	Continuous	Mean Difference	Fixed	12,867	− 1.02	− 1.42	− 0.63	49.07	< 0.00001	5.08	< 0.00001
BMI	3	Continuous	Mean Difference	Fixed	1987	0.75	0.22	1.28	0.36	0.83	2.77	0.006
Logistic EuroSCORE	4	Continuous	Mean Difference	Fixed	1739	− 0.27	− 1.68	1.15	0.59	0.90	0.37	0.71
STS score	2	Continuous	Mean Difference	Fixed	999	0.94	0.06	1.82	1.76	0.18	2.09	0.04
NYHA ≥ III	5	Dichotomous	Odds Ratio	Fixed	2288	0.86	0.65	1.13	2.74	0.6	1.09	0.28
MR ≥ 3 grade	3	Dichotomous	Odds Ratio	Fixed	1330	2.11	0.88	5.04	1.09	0.58	1.67	0.09
Hypertension	7	Dichotomous	Odds Ratio	Fixed	27,408	1.03	0.98	1.09	3.70	0.72	1.34	0.18
Diabetes mellitus	7	Dichotomous	Odds Ratio	Fixed	27,529	1.17	1.10	1.25	19.47	0.003	4.73	< 0.00001
Prior PCI	5	Dichotomous	Odds Ratio	Fixed	26,215	1.67	1.56	1.79	2.53	0.64	14.95	< 0.00001
Prior CABG	5	Dichotomous	Odds Ratio	Fixed	26,843	2.92	2.74	3.11	2.78	0.6	33.70	< 0.00001
Prior stroke	6	Dichotomous	Odds Ratio	Fixed	12,312	1.07	0.95	1.19	10.18	0.07	1.12	0.26
Coronary Artery disease	5	Dichotomous	Odds Ratio	Fixed	26,898	2.03	1.94	2.14	13.50	0.009	27.89	< 0.00001
COPD	5	Dichotomous	Odds Ratio	Fixed	2292	1.23	1.00	1.52	9.14	0.06	1.95	0.05
Renal failure	4	Dichotomous	Odds Ratio	Fixed	16,594	1.73	1.62	1.84	11.75	0.008	16.7	< 0.00001
Atrial Fibrillation	5	Dichotomous	Odds Ratio	Fixed	2305	1.01	0.85	1.20	5.61	0.23	0.08	0.94
Previous MI	6	Dichotomous	Odds Ratio	Fixed	2823	2.00	1.67	2.39	11.37	0.04	7.49	< 0.00001

### Risk of bias

The score of the scale is 0–9, and when the score is ≥ 7, it is considered to be a study with low risk. The risk deviation assessment was completed by two people independently, and when differences arose, they were resolved through discussion or negotiated by a third participant until agreement was reached. These figures were shown in Table 3.

**Table 3:** Results of NOS included in the study

Quality assessment									
Inclusion study	Selection			Comparability			Outcome		Total (minutes)
	①	②	③	④	⑤	⑥	⑦	⑧	
Attizzani	☆	☆	☆	☆	☆☆	☆	☆	/	8
Doshi	☆	☆	☆	☆	☆☆	☆	☆	☆	9
Elbadawi	☆	☆	☆	☆	☆☆	☆	☆	☆	9
Garfoor	☆	☆	☆	☆	☆☆	☆	☆	☆	9
Muhammad	☆	☆	☆	☆	☆☆	☆	☆	☆	9
Rodrigo	☆	☆	☆	☆	☆☆	☆	☆	☆	9
Tigger	☆	☆	☆	☆	☆☆	☆	☆	/	8
Werner	☆	☆	☆	☆	☆☆	☆	☆	/	8

①Representativeness of the Exposed Cohort

②Selection of the Non-Exposed Cohort

③Ascertainment of Exposure

④Demonstration That Outcome of Interest Was Not Present at Start of Study

⑤Comparability of Cohorts on the Basis of the Design or Analysis

⑥Assessment of Outcome

⑦Was Follow-Up Long Enough for Outcomes to Occur

⑧Adequacy of Follow Up of Cohorts

### Effect of gender in outcomes after TMVR

**Fig. 1:**
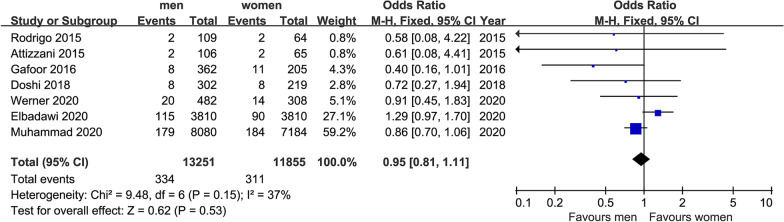
Flowchart of study selection for the present study

**Fig. 2:**
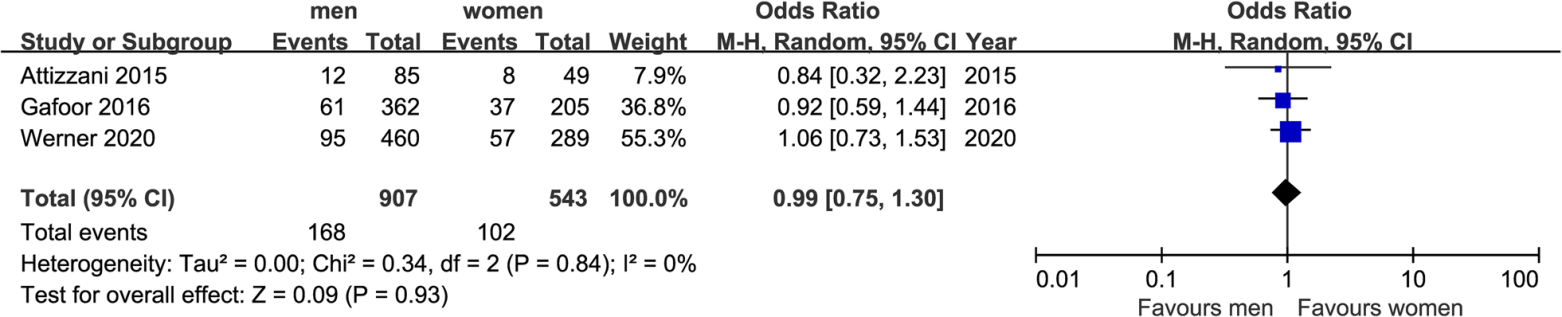
Forest plot of all-cause mortality (in 30 days)

**Fig. 3:**
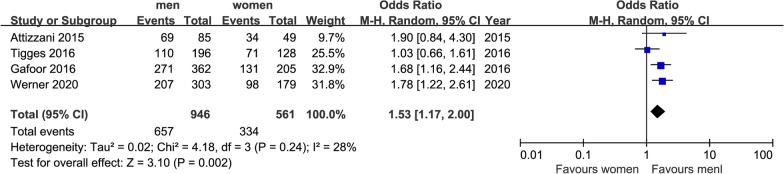
Forest plot of bleeding in hospital

**Fig. 4:**
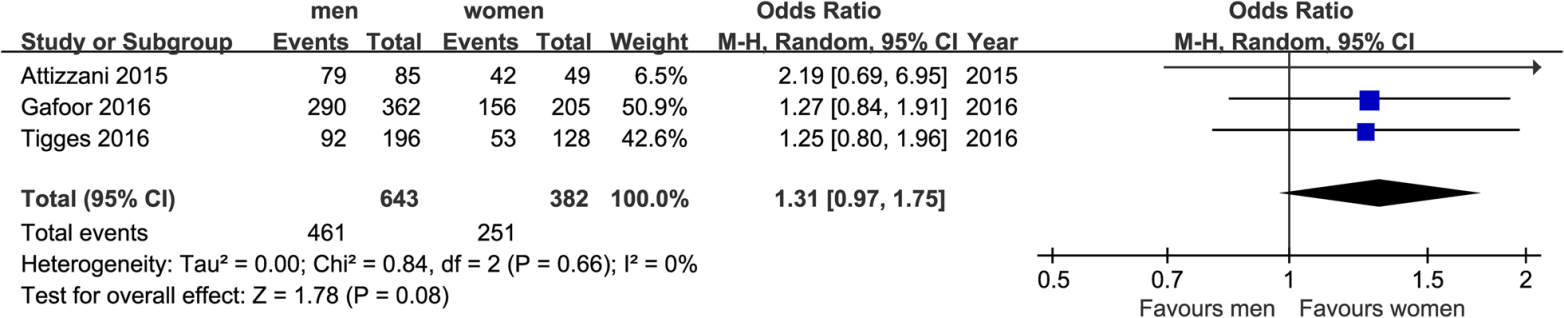
Forest plot of long-term mortality (in 2 years)

**Fig. 5:**
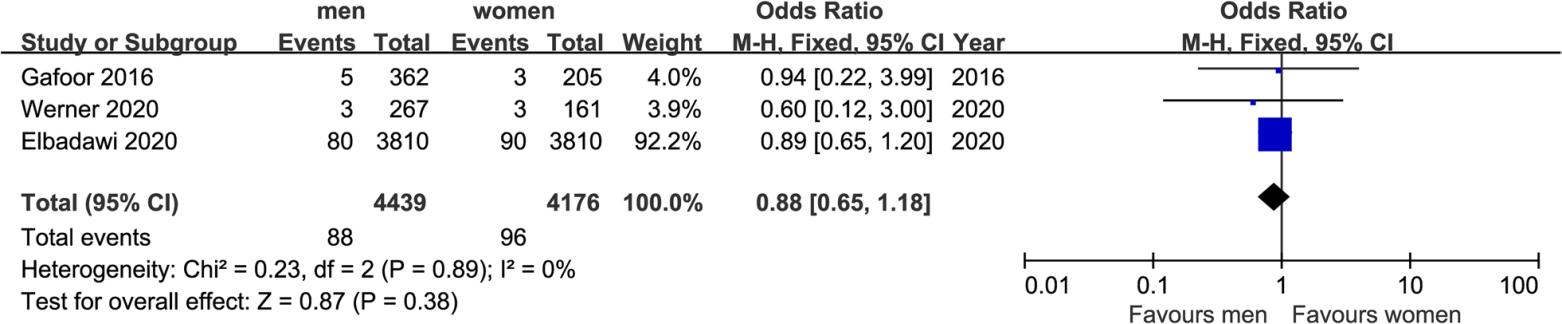
Forest plot of NYHA ≦ II class (after 1 year)

**Fig. 6:**
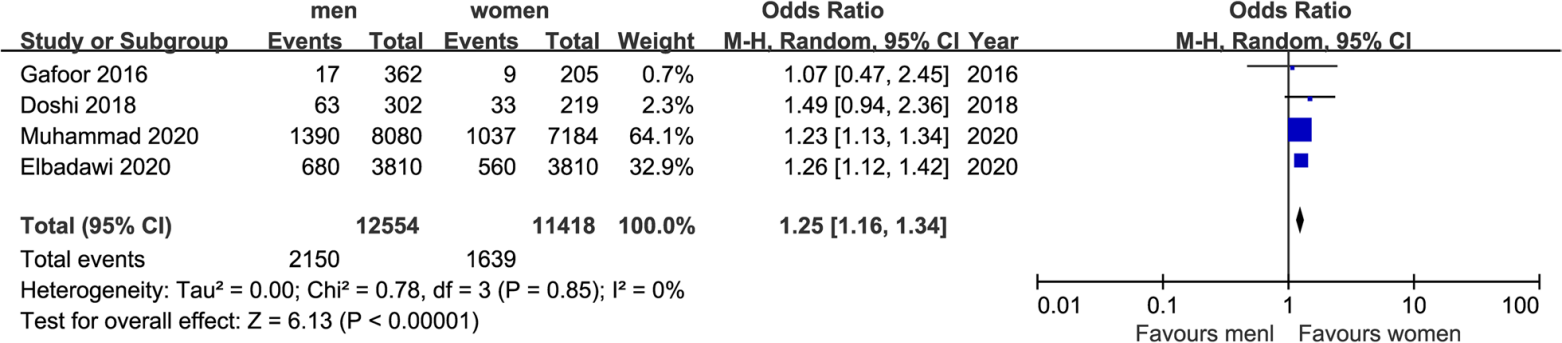
Forest plot of MR1-2 class (after 1 year)

**Fig. 7:**
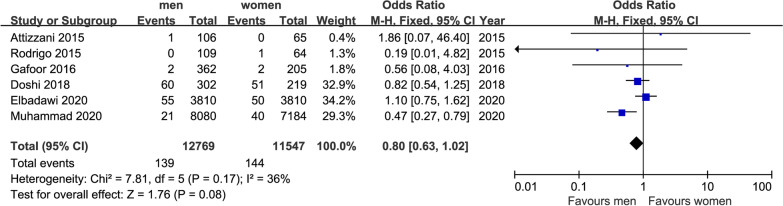
Forest plot of MI (after 1 year)

**Fig. 8:**
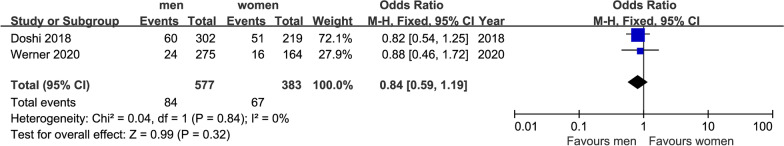
Forest plot of AKI (in 30 days)

We finally extracted mortality, NYHA class and some major complications as outcomes: short-term mortality (in 30 days) and long-term mortality (in 2 years), postoperative NYHA class (after 1 year), MR class (after 1 year), stroke (in 30 days), MI (after 1 year), acute kidney injury (AKI) (in 30 days) and bleeding in hospital. After our analysis, only the differences of postoperative NYHA class (after 1 year) and incidence of AKI (in 30 days) made sense. All forest plots were shown in Figs. 1, 2, 3, 4, 5, 6, 7, 8. We see no heterogeneity among all outcomes after TMVR.

#### Mortality

##### Short term mortality (in 30 days)

We identified seven studies that reported gender difference of all-cause mortality after TMVR in 30 days; Data on 13,251 men and 11,855 women were available for analysis, the mortality was 2.5% in men and 2.6% in women. As shown in Fig. 1, there were no significant gender differences for this outcome (OR = 0.95, 95%CI [0.81,1.11], P = 0.53). No indication of heterogeneity between the results of the different trials was presented (Q = 9.48, DF = 6, P = 0.15, I^2^ = 37%).

##### Long-term mortality (in 2 years)

Three studies were eligible for gender difference in long-term mortality. These studies enrolled 907 men and 543 women, the mortality was 18.5% in men and 18.8% in women. As shown in Fig. 2, there were no significant differences for this mortality (OR = 0.99, 95%CI [0.75,1.30], P = 0.93). There was no indication of heterogeneity between the results of the different trials (Q = 0.34, DF = 2, P = 0.84, I^2^ = 0%).

#### Postoperative heart-related indexes

##### NYHA (after 1 year)

We got this index from four studies, 946 men and 561 women were participated, in which the incidence of NYHA ≦ II class was 69.5% in men and 59.5% in women respectively. Although the lengths of the observation periods differ among the respective studies, the advantage of men’s NYHA was presented and corresponded to above 1.5 times to women (OR = 1.53, 95%CI [1.23,1.91], P = 0.0001), which is shown in Fig. 3. No indication of heterogeneity between the results of the different trials was presented (Q = 4.18, DF = 3, P = 0.24, I^2^ = 28%).

##### MR1-2 class (after 1 year)

Number of studies reported MR1-2 class after 1 year were only three. For this index, there were 643 men and 382 women. The incidence of MR1-2 class was 71.8% in men and 65.7% in women. It is shown that there is no significant difference for MR1-2 class in Fig. 4 (OR = 1.30, 95%CI [0.97,1.75], P = 0.08). There was no indication of heterogeneity between the results of the different trials (Q = 0.84, DF = 2, P = 0.66, I^2^ = 0%).

#### Complications

##### MI (after 1 year)

With regarded to the incidence of MI after TMVR for a year, there were 184 events in 8515 patients in three studies. As shown in Fig. 5, there was no significant difference between the results of MI (OR = 0.88, 95%CI [0.65, 1.18], P = 0.38). No indication of heterogeneity between the results of the different trials was presented (Q = 0.23, DF = 2, P = 0.89, I^2^ = 0%).

##### AKI (in 30 days)

A total of six studies including 23,972 patients (12,544 men and 11,478 women) provided data on incidence of AKI in 30 days. Meta-analysis shown 1.25 times in this complication (OR = 1.25, 95%CI [1.16,1.34], P < 0.05) in Fig. 6, which corresponded that men had worse renal outcome than women. There was no indication of heterogeneity between the results of the different trials (Q = 0.78, DF = 3, P = 0.85, I^2^ = 0%).

##### Stroke (in 30 days)

The incidence of stroke after TMVR in 30 days was reported in six studies. Although there was a slight tendency toward a higher rate in women, this was not significant (139/12769 in men and 144/11547 in women), which is shown in Fig. 7 (OR = 0.80, 95%CI [0.63,1.02], P = 0.08). No indication of heterogeneity between the results of the different trials was presented (Q = 7.81, DF = 5, P = 0.17, I^2^ = 36%).

##### Bleeding in hospital

**Fig. 9:**
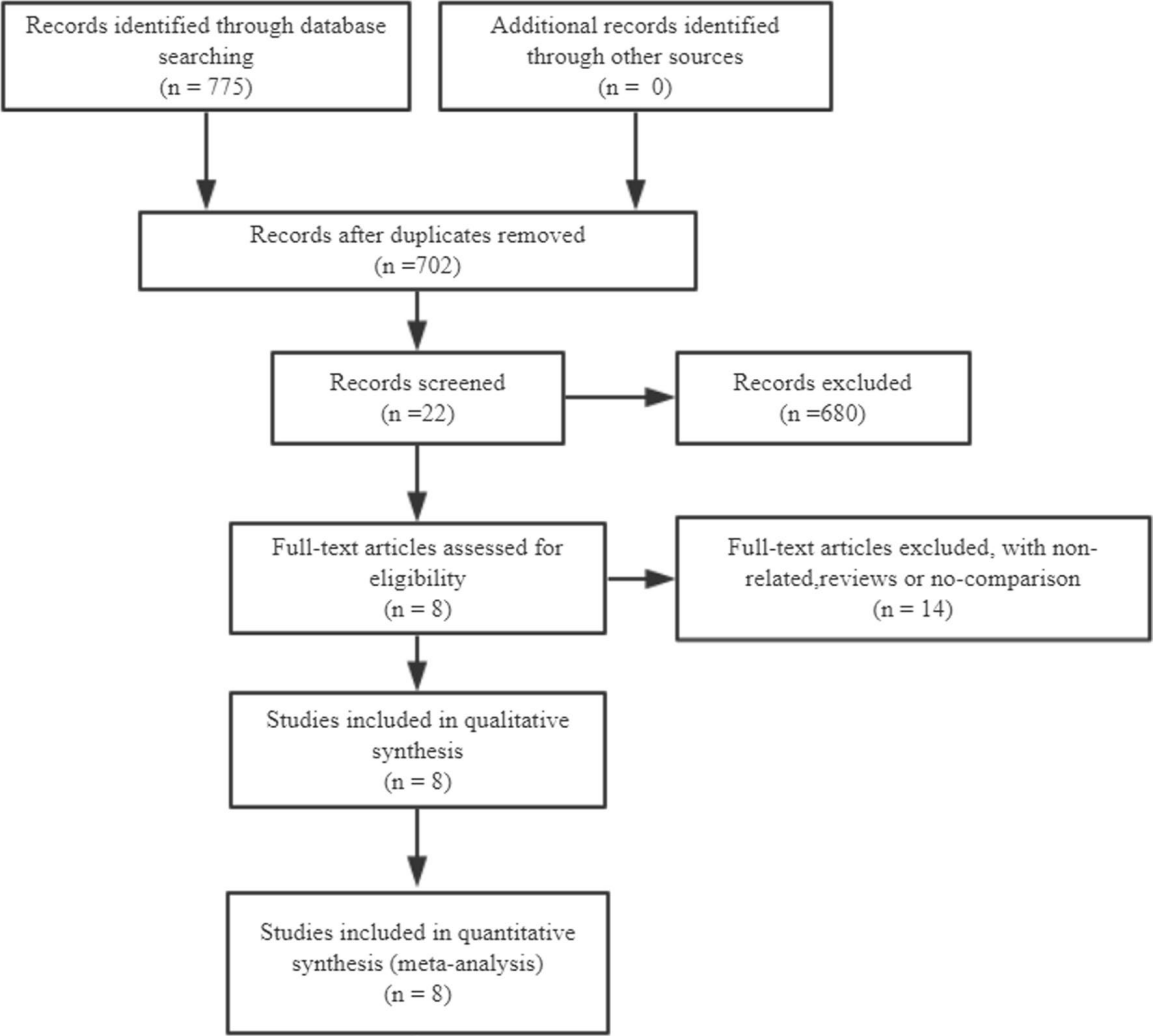
Forest plot of stroke (in 30 days)

Two studies involving 960 patients reported data on incidence of bleeding in hospital. There were 84 out of 577 men and 67 out of 383 women, as shown in Fig. 8, there were no significant difference of this complication based on gender (OR = 0.84, 95%CI [0.59,1.19], P = 0.32). There was no indication of heterogeneity between the results of the different trials (Q = 0.04, DF = 1, P = 0.84, I^2^ = 0%) (Fig. 9).

## Discussion

With the expansion of indications for TMVR, it has become a good choice for many patients who are not suitable for surgical repair. With the progress of systems for MitraClip, MitraClip G4 system has been launched [34]. Many problems of TMVR are still being studied. The efficacy of TMVR compared with traditional surgical repair has also been confirmed by many experiments. In this paper, a meta-analysis was conducted on the gender-related difference of prognosis, with a view to providing certain significance for clinical treatment.

The meta-analysis showed that the incidence of the underlying diseases (diabetes mellitus, coronary artery disease, renal failure and myocardial infarction) was higher in men than in women before TMVR, and MR class was higher. There was no significant difference in the NYHA class between the two groups. On this basis, there was no significant difference in the incidence of short-term and long-term mortality and other complications between men and women after TMVR, except for AKI, which is higher in men than women. Men have more preoperative renal failures and they have a higher incidence of postoperative AKI than women. This is consistent with the baseline results. MR class and NYHA class were improved, which were more obvious in men. The average age of men is younger than that of women, which may be one of the reasons.

Gender difference is an inherent character in studying a group of people. It is precisely because of gender difference that men and women are inevitably distinguished from each other at the beginning of receiving a certain operation type. In our included studies, gender-difference is no more recognized as a baseline index, all patients were divided into different groups according to gender difference. If there is a difference in an index after receiving a certain TMVR which is opposite to the preoperative baseline, it is more likely to support the important role of gender difference in this index. Among the above indicators, NYHA class is one of them. There was no significant difference of preoperative NYHA class between men and women, but men had a higher proportion of preoperative MI and a higher proportion of preoperative PCI and CABG history than women. However, postoperative NYHA class of men showed better improvement than that of women, and the proportion of patients with postoperative NYHA function rating of 1–2 was higher than that of women, which meant that men had a better prognosis than women. There were more underlying diseases and surgical history in men than in women, which may be related to the higher incidence of postoperative AKI. In addition, there was no significant difference in the incidence of stroke before and after surgery between men and women, and no significant difference in short and long-term mortality.

Snah thought that the postoperative differences between men and women came from their anatomical differences actually [35]. For example, women have smaller ventricular cavity, and even with body surface area as an indicator, the left ventricular volume of women is still smaller. If women have MR, the symptoms are more typical. In addition, the annulus area, the distance between papillary muscles and the effective mitral regurgitation area (EROA) were smaller. The higher ratio of EROA to left ventricular end diastolic volume (LVEDV) suggests that MR is more severe [36]. Mantovani advanced that there were significant differences between men and women undergoing MR surgery [23]. Due to the lower EROA, some women are less classified as severe MR, for these women who received TMVR, they had better health condition than men, so there is little difference before and after TMVR, which can explain why men have better improvement in MR and NYHA class on the basis of worse preoperative evaluation. And some women who are more critical and are not suitable for thoracotomy repair have more serious reflux, so even if they are treated with TMVR, the improvement effect is not as obvious as that of male patients. Although men have more basic diseases before operation, their MR status and NYHA class are not as serious aswomen, so they have better improvement. There are certain differences in the proportion of men and women in the number of TMVR: in the studies included in this meta-analysis, men have a higher proportion of TMVR (59%), and women have more open chest repair. There is no clear report on the difference of MR incidence between male and female. In the study of Shah [35], Grayburn [36] and Lampert [37], the ratio of EROA to LVEDV may be a better manifestation of MR, which can better represent the patient's MR status to a certain extent, so as to better guide clinical treatment.

It is estimated that 2% of the global population displays symptoms of mitral valve (MV) disease [3], and early treatment is extremely important. TMVR is a minimally invasive and effective method, and its research is of great significance. As far as the difference between men and women is concerned, with the progress of technology, more and more patients will perform this operation. The results shown in this paper need more data support to further clarify.

This paper still has some limitations: (1) Literatures included in this study were all cohort studies. According to the new Level 5 standard of Oxford University EBM Center on literature types, the included literature evidence level was 2B (cohort studies or poor randomized controlled studies), which may affect the strength of the argument; However, gender cannot be randomized. (2) Clinical, anatomical and physiological differences between men and women cannot be avoided. (3) The further gender-related prognosis cannot be assessed because the lack of includes studies. (4) The including studies and the number of patients were too few to make a subgroup analysis, like the type of MR; (5) Some data came from the database, although the effect on the results was not significant.

## Conclusions

Our study demonstrates that there are no major gender-related differences concerning (1) short- term and long-term mortality. (2) the incidence of postoperative stroke, MI and bleeding in hospital. However, men gender is associated with superior postoperative NYHA class. As a result, whether it is men or women, it is important to address clinical, anatomical and physiological sex-related differences to clarify the discrepancy of the prognosis after TMVR.

## Data Availability

All data generated or analysed during this study are included in this published article.
